# Significance of honeybee pollination in increasing seed yield of *Trifolium alexandrinum* (Fabales: Fabaceae) and its impact on economic sustainability of smallholder farmers

**DOI:** 10.1093/jee/toae222

**Published:** 2024-10-11

**Authors:** Muhammad Shoaib Tufail, Gaye L Krebs, Alison Southwell, John W Piltz, Peter C Wynn, David F Cook

**Affiliations:** Department of Primary Industries and Regional Development, Horticulture and Irrigated Agriculture, South Perth, Western Australia 6151, Australia; Gulbali Institute of Agriculture, Water and Environment, Charles Sturt University, Wagga Wagga, New South Wales 2678, Australia; Gulbali Institute of Agriculture, Water and Environment, Charles Sturt University, Wagga Wagga, New South Wales 2678, Australia; School of Agricultural, Environmental and Veterinary Sciences, Charles Sturt University, Wagga Wagga, New South Wales 2678, Australia; Gulbali Institute of Agriculture, Water and Environment, Charles Sturt University, Wagga Wagga, New South Wales 2678, Australia; School of Agricultural, Environmental and Veterinary Sciences, Charles Sturt University, Wagga Wagga, New South Wales 2678, Australia; Wagga Wagga Agricultural Institute, Department of Primary Industries, Wagga Wagga, New South Wales 2650, Australia; Gulbali Institute of Agriculture, Water and Environment, Charles Sturt University, Wagga Wagga, New South Wales 2678, Australia; Department of Primary Industries and Regional Development, Horticulture and Irrigated Agriculture, South Perth, Western Australia 6151, Australia

**Keywords:** bees pollination, berseem clover, forage seed production, insect economics, sustainable yields, on-farm profitability

## Abstract

A major limitation to producing high seed yields in berseem clover (*Trifolium alexandrinum* L.) is failure to set seed, predominantly due to lack of pollination. Despite the importance of berseem clover as a leading forage legume, the contribution of pollinators to seed set is scarce. In Pakistan, the honeybee population is declining mainly because of the extensive use of neonicotinoid pesticides and habitat fragmentation. This, combined with the region’s harsh environment and the use of inferior, locally bred genotypes, has resulted in low seed yields. Insufficient seed availability leads to limited forage supply, resulting in poor livestock nutrition, which subsequently impacts livestock health and productivity, and reduces farmers’ income. The present study estimated the seed production of 3 berseem clover genotypes resulting from honeybees [*Apis mellifera* L. (Hymenoptera: Apidae)] pollination in 2 growing seasons (2012–2014) in the central Punjab region of Pakistan. Experiments had 2 pollination treatments (open pollination and honeybee inclusion) and 3 seed genotypes, *viz.* farmer own-saved, market, and the improved variety cultivars. For both growing seasons, honeybee pollination resulted in significant increases in seed yields ranging from 35% to 67%, regardless of seed genotype. With the exception of the number of seed heads/m^2^, all seed yield parameters also increased significantly in response to honeybee inclusion. The combination of improved variety and honeybee inclusion resulted in the production of a maximum number of seeds per head (45.3), 1,000-seed weight (3.7 g), and estimated seed yield (375.5 kg/ha). In addition, the increase in estimated net income of seed ranged from PKR 82,485 Rs/ha (US$844/ha) to PKR 168,975 Rs/ha (US$1728/ha) with the use of honeybees as an insect pollinator across all the seed genotypes. Honeybee pollination has broader implications for mixed farming systems by playing a key role in preserving biodiversity and promoting sustainable agriculture. It also enhances the quality and quantity of berseem crops by increasing the production of high-quality seeds and forage leading to improved livestock productivity and family food security which strengthens the economic resilience of rural communities.

## Introduction

Seed production in various grains, horticulture, and forage crops is closely related to the activity of insect pollinators, with honeybees (*Apis* spp.) considered to be the primary insect pollinator. Honeybees [*Apis mellifera* L. (Hymenoptera: Apidae)] contribute to the pollination of about 80% of all major seed-producing crops (Meena et al. 2015, Cook et al. 2023). In Pakistan, berseem clover (*Trifolium alexandrinum* L.) is a very popular forage crop amongst farming communities grown by 94% of farmers (Ul-Allah et al. 2014), and thus the most widely grown winter forage legume, however, seed shortage during the growing season has substantially reduced its productivity (Sarwar et al. 2002). Forage legumes are protein-rich plants cultivated to produce nutritious animal feed, particularly for grazing or as harvested fodder, and to enhance soil health and fertility by fixing atmospheric nitrogen into the soil (Tufail et al. 2020).

Formal seed supply systems often neglect forages, leading to a gap between seed demand and supply, and provide only 10% of the basic seed requirement while the rest is obtained from informal systems, primarily through farmers’ own-saved seed or farmer-to-farmer exchange (Bishaw and Turner 2008). This farmer-produced seed has the advantages of being economical, of better quality, and is readily available at the time of sowing. Berseem clover, a vital forage crop in mixed farming systems, addresses forage and seed shortages while enhancing soil fertility (through nitrogen fixation), conserving natural resources, and promoting sustainable crop-livestock production (Tufail et al. 2020). Facilitating honeybee pollination significantly boosts seed yields, improving forage availability and livestock productivity. However, inadequate pollination remains a key limiting factor in achieving higher and more reliable seed yields from berseem clover (Sanjay Kumar et al. 2021, Harris and Ratnieks 2024).

Berseem clover is a self-pollinated, forage crop and produces self-sterile flowers, however, these tend to be cross-pollinated up to 82% by different insect pollinators (Bakheit 1989). Berseem clover flowers have a simple valvular type mechanism of pollination, and for this, the tripping mechanism (release of the staminal column on the wings of the insect and to the keel petals of flowers) is very important (Roy et al. 2005). Rauf et al. (2021) noted that in alfalfa (*Medicago sativa* L.)—another forage legume, solitary bees like *Megachile cephalotes*, *Megachile hera*, and *Amegilla* species were identified as the most effective pollinators for seed production. Insects play a crucial role in this tripping mechanism, especially honeybees (Latif et al. 2014), as they have been found to be the most abundant (insect) visitors to berseem flowers (Dixit et al. 1989, Jat et al. 2014b). Moreover, crop genetics, climate, insect population, and forage harvest times (especially last fodder cut) affect both flowering and seed setting, and ultimately impact the seed yield of berseem clover (Tufail et al. 2020). Furthermore, the environmental conditions (temperature, rainfall, and humidity) not only have a direct effect on seed yields but also can influence the movement of honeybees as pollinators (El-Naby et al. 2012), thereby impacting pollination efficiency and subsequent seed set. However, environmental conditions are not the only factors impacting honeybees.

In Pakistan, the beneficial insect populations, especially native honeybee populations, are declining rapidly (Murray et al. 2009). Between 2007 and 2016, the honeybee population declined by 40% and food production (resulting from pollination) declined by 33% (Ahmad and Aziz 2017). These declines can be attributed to the intensive and improper use of neonicotinoid pesticides (Ahmed et al. 2014) and the impact of viral diseases (Amjad Ullah et al. 2021), resulting in low honeybee populations as well as a reduction in nesting sites due to habitat destruction and fragmentation (Latif et al. 2014). In the central Punjab region of Pakistan, the insect taxa commonly visiting berseem clover crop as pollinators include native honeybees such as *A. dorsata* (rock honeybee), *A. florea* (dwarf honeybee), and *A. cerana* (Asian hive honeybee), along with non-native honeybees like *A. mellifera* (European honeybee). In addition, frequent pollinators of berseem crop are the ladybird (*Coccinella septempunctata*), hoverfly (*Episyrphus balteatus*), bumblebee (*Bombus haemorrhoidalis*), syrphid fly (*Eristalinus aeneus*), and butterflies such as *Belenois aurota*, *Junonia orithya*, and *Danaus plexippus* (Ahmad and Aziz 2017, Suneela et al. 2023). Farmers need to be more aware of the importance of pollinators (particularly honeybees) in agricultural production systems to ensure responsible farming practices, such as avoiding the use of neonicotinoid chemicals. In addition, promoting sustainable agricultural practices, such as cultivating legume forage crops, is essential for reducing the negative impacts of food production on the ecosystems and maintaining or enhancing the overall productivity of these systems (Harris and Ratnieks 2024).

Despite all these factors, the increased presence of a pollinator population is needed for maximizing seed yield in berseem crop. Roy et al. (2005) found hand tripping and controlled honeybees pollination visits by placing honeybees in enclosed pasture plots improved seed setting in berseem clover when compared to normal open pollination. They also found that the exclusion of pollinators by enclosing pasture reduced seed setting between 12% and 99% relative to open pollination across all varieties, even in self-pollinating lines of berseem clover. Similarly, Rodet et al. (1998) found that the honeybees increased the process of pollination and fertilization by up to 90% which contributed significantly to white clover (*Trifolium repens* L.) seed yield in the Mediterranean conditions. However, no studies have been undertaken in Pakistan to determine the honeybee pollination impact on berseem clover seed production.

Therefore, the aim of this research was to determine the effect of honeybee pollination on berseem clover seed yield originating from farmer own-saved, market, and research-station seed cultivars. In addition, the potential impact on financial returns to smallholder farmers growing seed on a commercial basis in Pakistan was also investigated in this study.

## Materials and Methods

For 2 successive growing seasons (2012–2013 and 2013–2014), field trials were undertaken to determine the effects of honeybee pollination on the seed yield parameters including number of seed heads per m^2^, number of seeds per head, 1,000-seed weight (g), and estimated seed yield (kg/ha) of 3 different berseem clover genotypes at the University of Veterinary and Animal Sciences (UVAS), Ravi-Campus Pattoki in district Kasur, Pakistan (31°03’35‘ N, 73°52’42’ E, altitude 218 m). The study was approved by the Charles Sturt University’s Human Research Ethics Committee (Protocol # 416/2012/12).

### Experimental Design

A randomized block design with 2 treatment factors (pollination and genotype) in a split-plot arrangement was used, having 3 replications. A plot size of 7 m × 3 m (21 m^2^) was used for each treatment combination. The pollination treatment factor was randomized to the whole-plot level to enable honeybees to be kept within one inclusion zone, while the genotype factor was randomized to the sub-plot level (Table 1). The 3 genotypes of berseem clover were farmer own-saved seed (LBF1), market seed (LBM1), and the improved variety (cv. Agaitti Berseem-2002; AB-2002) seed. The improved variety was bred locally at the Fodder Research Institute, Sargodha, Pakistan; the farmer seed was grown and retained on-farm by local farmers (as local landraces); and the market seed was sold locally in the agricultural market (mainly consisted of low-quality imported varieties).

**Table 1.** Layout of experimental design using 3 genotypes of berseem clover: farmer own-saved (LBF1), market (LBM1), and research-station (cv. Agaitti Berseem-2002; AB-2002) seeds in combination with the respective treatment of honeybee inclusion (BI) and open pollination (OP)

Replications	Honeybee inclusion (BI) treatments (Netted area)^*^	Open pollination (OP) treatments (Open area)
1	Market seed × Bee inclusion (LBM1 × BI)	Farmer own-saved seed × Open pollination (LBF1 × OP)
	Farmer own-saved seed × Bee inclusion (LBF1 × BI)	Research-station seed × Open pollination (AB-2002 × OP)
	Research-station seed × Bee inclusion (AB-2002 × BI)	Market seed × Open pollination (LBM1 × OP)
2	Farmer own-saved seed × Bee inclusion (LBF1 × BI)	Market seed × Open pollination (LBM1 × OP)
	Market seed × Bee inclusion (LBM1 × BI)	Research-station seed × Open pollination (AB-2002 × OP)
	Research-station seed × Bee inclusion (AB-2002 × BI)	Farmer own-saved seed × Open pollination (LBF1 × OP)
3	Research-station seed × Bee inclusion (AB-2002 × BI)	Research-station seed × Open pollination (AB-2002 × OP)
	Farmer own-saved seed × Bee inclusion (LBF1 × BI)	Farmer own-saved seed × Open pollination (LBF1 × OP)
	Market seed × Bee inclusion (LBM1 × BI)	Market seed × Open pollination (LBM1 × OP)

^*^Honeybee inclusion (BI) treatment plots were netted together under 1 large nylon net with a total area of 189 m^2^.

The 2 pollination treatments were open pollination (OP—natural pollination levels for the district) and honeybee inclusion (BI—honeybees introduced and retained on-site to maximize honeybee population and pollination rates). Honeybee inclusion was chosen (as opposed to honeybee exclusion) as it was anticipated that honeybee populations for the district were low. Furthermore, honeybee inclusion would provide a comparison between current and potential pollination rates between the various berseem clover genotypes used in the present study.

### Agronomic Practices

Preparation of the field sites involved 3 ploughings using a cultivator followed by 2 plankings with a wooden planker (locally named as Suhaga). Nitrogen (N), phosphorus (P_2_O_5_), and potassium (K_2_O) fertilizers were applied at the locally recommended rates of 20, 60, and 30 kg/ha, respectively; using urea (46% N), di-ammonium phosphate (DAP; 18% N and 46% P_2_O_5_), and muriate of potash (MOP; 60% K_2_O) fertilizers. All the fertilizers were broadcast by hand and then incorporated into the soil prior to sowing. Seed from each genotype was cleaned manually and inoculated with *Rhizobium trifolii* prior to sowing. Following land preparation, the plots were flood irrigated and seeds were hand-broadcasted during the last week of October at the recommended seed rate of 20 kg/ha. The first irrigation was applied 1 week after sowing, with subsequent irrigations every 2 weeks based on rainfall during the 2012–2013 growing season. In contrast, the following season (2013–2014) received below-average rainfall, indicating drier conditions. To compensate, additional and more frequent flood irrigations (after every 7–10 days) were applied to the berseem clover to maintain adequate soil moisture and support crop growth. Three forage cuts were taken at 65, 110, and 150 days (d) after sowing before the crop was left for seed production (Tufail et al. 2020).

**Fig. 1.**
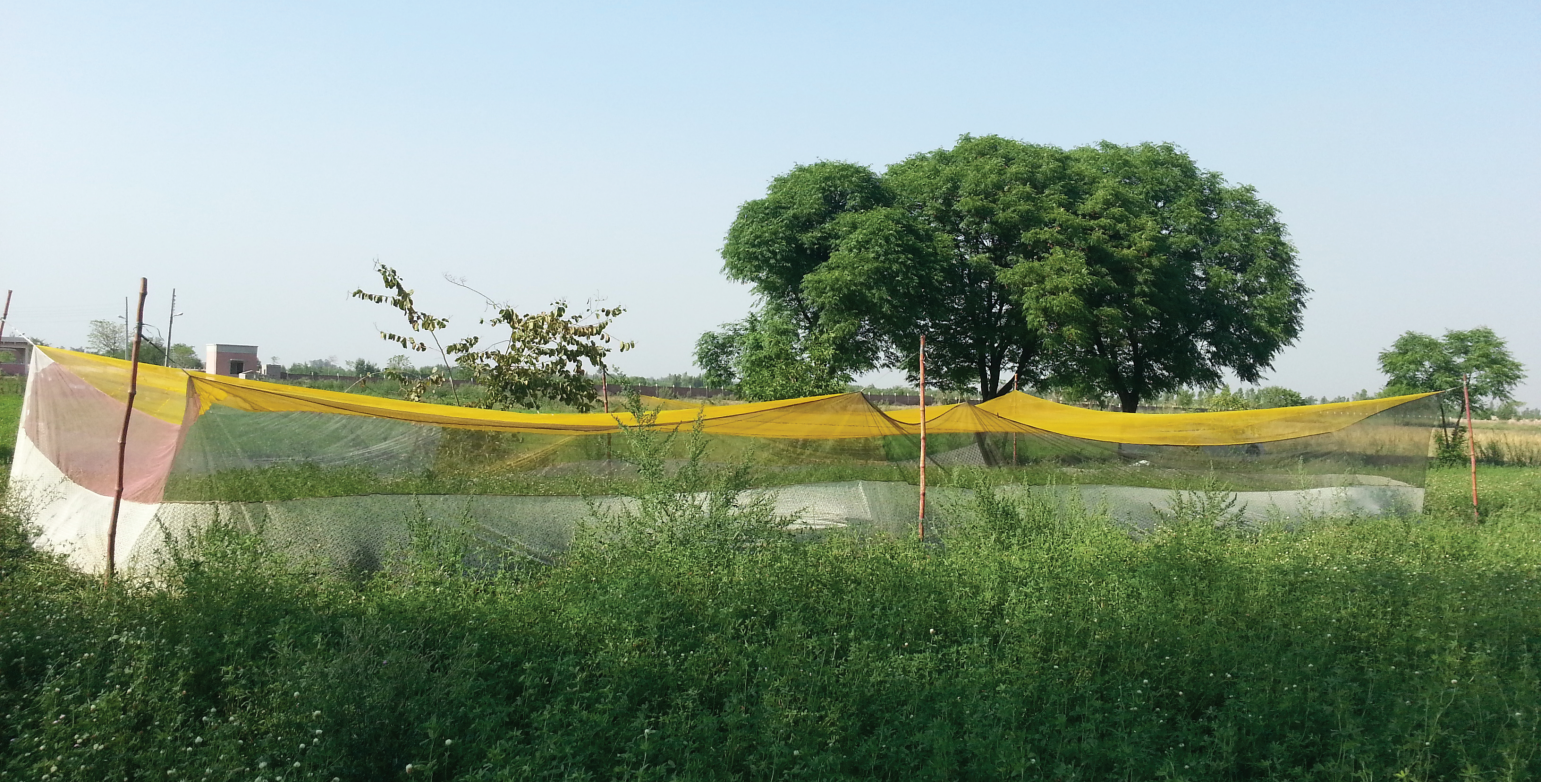
Field trial plots netted area for the bee pollination experiment to allow honeybee pollination of berseem clover at the Ravi-campus of the University of Veterinary and Animal Sciences (UVAS) Pattoki located in the district Kasur of Punjab, Pakistan.

After the final forage harvest (in mid-March), all the honeybee inclusion plots were netted together (an area of 189 m^2^) using a nylon net. The 3-m-high nets were constructed to allow unhindered movement of honeybees inside, while at the same time excluding the entry of other insects. The nets were erected with the help of bamboo poles and tightened using nylon cords. The edge of the netting was sufficiently pressed into the soil to prevent movement of insects and honeybees in and out of the netted plots (Fig. 1). A zipped entry was kept at one corner of the net to allow manual entry for data recording and other operations. A colony of honeybees in standard wooden hives containing approximately 7,000–10,000 honeybees (sourced from a private contractor) was introduced into the netted area at the start of flowering. The colony was regularly provided with access to water and a sugar solution (molasses). The colony was removed at the end of flowering when the pollination process was completed.

### Sampling, Data Collection, and Estimation of Net Incomes

At the time of seed harvest, within each plot, 3 areas (1 m^2^ quadrat) were randomly selected, avoiding the edges in each plot. The number of seed heads was counted and then averaged. Ten seed heads were then randomly selected from each plot, the seeds were removed, counted, and the average number of seeds per head was calculated. The 1,000-seed weight (g) was determined by counting out one thousand seeds (for each treatment) and then weighing them using an electric weighing balance. The predicted seed yields were then calculated by multiplying the number of heads per m^2^, number of seeds per head and 1,000-seed weight, and then converted to kg/ha. The percent increase in seed yield with honeybee pollination over open pollination was calculated by using the following formula:


$$\begin{array}{lll} & Percent\;increase\;\left(\%\right)=\; & \frac{Bee\;inclusion\;seed\;yield-Open\;pollination\;seed\;yield}{Open\;pollination\;seed\;yield}\;\;\times \;100 \end{array}$$

In addition to the seed yield parameters calculations, the net income from seed in Pakistani rupees (PKR) was also calculated by multiplying the weight of seed produced (kg) by the prevailing market price for berseem clover seed, which was PKR 450 Rs/kg. These figures were then converted to American dollars (US$) divided by the 5-year average of US$ to PKR exchange rate (US$1 = 97.76 PKR) (Oanda 2016).

### Statistical Analysis

The data collected for berseem clover seed yield parameters, including the number of seed heads per m^2^, number of seeds per head, 1,000-seed weight (g), and estimated seed yield (kg/ha), were analyzed using the GenStat statistical software. A linear mixed model (Asreml) was employed, with seed sources/genotype (SS), pollination treatment (PT), and year (Y) considered as fixed effects, and the replications (Rep), whole-plot (Wplot), and sub-plot (Splot) treated as random effects in the model (VSN International 2014). The statistical model equation is as follows:


$$\begin{array}{llllll} Responsevariable=\; & Constant+SS+PT+Y\; & +\left(SS\times PT\right)+\left(SS\times Y\right)+\left(PT\times Y\right)\; & +\left(SS\times PT\times Y\right)\; & +Rep+\left(Rep\times Wplot\right)\; & +\left(Rep\times Wplot\times Splot\right) \end{array}$$

The statistical model facilitates the assessment of the interactions between genotypes, pollination treatments, and year. The treatment means were then compared with the least significant differences at a 5% level of significance by using Tuckey’s HSD tested at *P* < 0.05, and the data are presented as the mean ± the standard error of the mean.

## Results

**Fig. 2.**
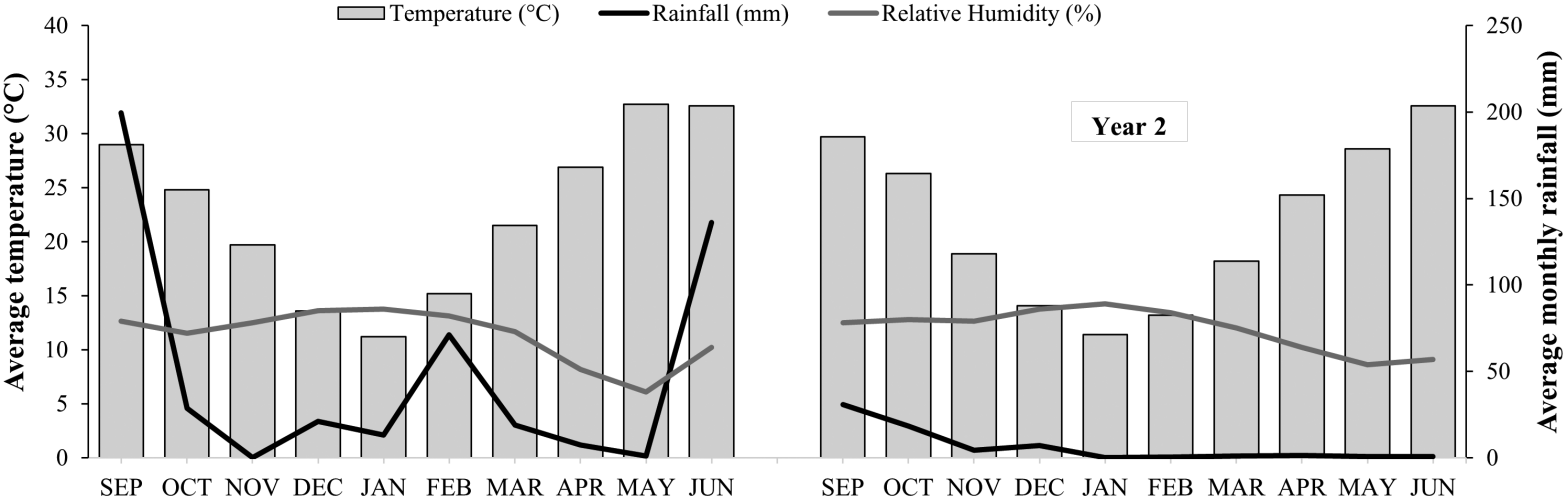
Average rainfall (mm), relative humidity (%), and temperatures (°C) per month during 2012–2013 and 2013–2014 growing seasons (September to June) in Pattoki, district Kasur, Pakistan.

The meteorological data for temperature, rainfall, and relative humidity for the study location for the 2 years of the study are presented in Fig. 2. Both average rainfall and relative humidity were high in season one (2012–2013) as compared to season 2 (2013–2014). However, there was little difference in the recorded temperature over the 2 seasons.

The effect of the honeybee inclusion on average berseem clover seed yield parameters including estimated seed yield (over 2 growing seasons) is presented in Table 2. Within each pollination treatment, the number of seeds per head was higher for the improved variety ‘Agaitti Berseem-2002’ (39.9 ± 2.2) compared to farmer seed (30.4 ± 2.0) or market (33.7 ± 2.1) seeds (*F* = 15.25; df = 3, 44; *P* < 0.001). The highest number of seeds per head (45.3) was produced using the improved variety and honeybee inclusion (AB-2002 ×  BI). However, the number of seed heads per m^2^ did not vary between the different sources of seed (230.7 vs. 218.5 vs. 201.6) or pollination treatments (212.3 vs. 221.5) or year (206.7 vs. 227.2) (*F* = 0.96; df = 3, 44; *P* = 0.418). There was no interaction (*P* > 0.05) found between pollination treatment (honeybee inclusion) and genotypes (seed sources) for any of the seed yield parameters measured in this study (Table 2).

**Table 2.** Effect of honeybee inclusion (honeybee pollination; BI) and open pollination (OP) on seed yield parameters (± SED) investigated with 3 different berseem clover genotypes/seed sources: farmer own-saved (LBF1), market (LBM1), and research-station (cv. Agaitti Berseem-2002; AB-2002). A linear mixed model (Asreml) was employed, with seed sources/genotype and pollination treatment as fixed effects, while the replications, whole-plot and sub-plot treated as random effects

Parameters	LBF1		LBM1		AB-2002		*P* value (± SED)		
	OP	BI	OP	BI	OP	BI	PT	SS	PT × SS
Seed heads per m^2^	204.4 (± 16.3)	232.6 (± 11.5)	203.1 (± 18.0)	200.0 (± 15.0)	229.5 (± 22.4)	232.0 (± 25.6)	0.120	0.209	0.087 (± 24.4)
Seeds per head	25.65^a^ (± 1.7)	35.25^b^ (± 3.0)	28.85^a^ (± 2.3)	38.60^b^ (± 2.5)	34.48^b^ (± 1.4)	45.30^c^ (± 3.1)	0.003	<0.001	0.767 (± 2.6)
1,000-seed weight (g)	2.135^a^ (± 0.19)	2.371^b^ (± 0.27)	2.243^ab^ (± 0.11)	2.375^b^ (± 0.25)	3.398^c^ (± 0.08)	3.676^d^ (± 0.06)	0.013	<0.001	0.985 (± 0.2)
Estimated seed yield (kg/ha)	110.8^a^ (± 13.0)	185.5^b^ (± 15.8)	135.6^a^ (± 23.5)	183.3^b^ (± 25.5)	272.4^c^ (± 33.8)	375.5^d^ (± 34.2)	<0.001	<0.001	0.552 (± 34.9)

OP = open pollination, BI = inclusion of honeybees, PT = pollination type, SS = seed source/genotypes.

SED = Standard error of differences (values within parenthesis are representing SED values).

Values within rows with varying superscripts differ significantly (*P *< 0.05).

**Fig. 3.**
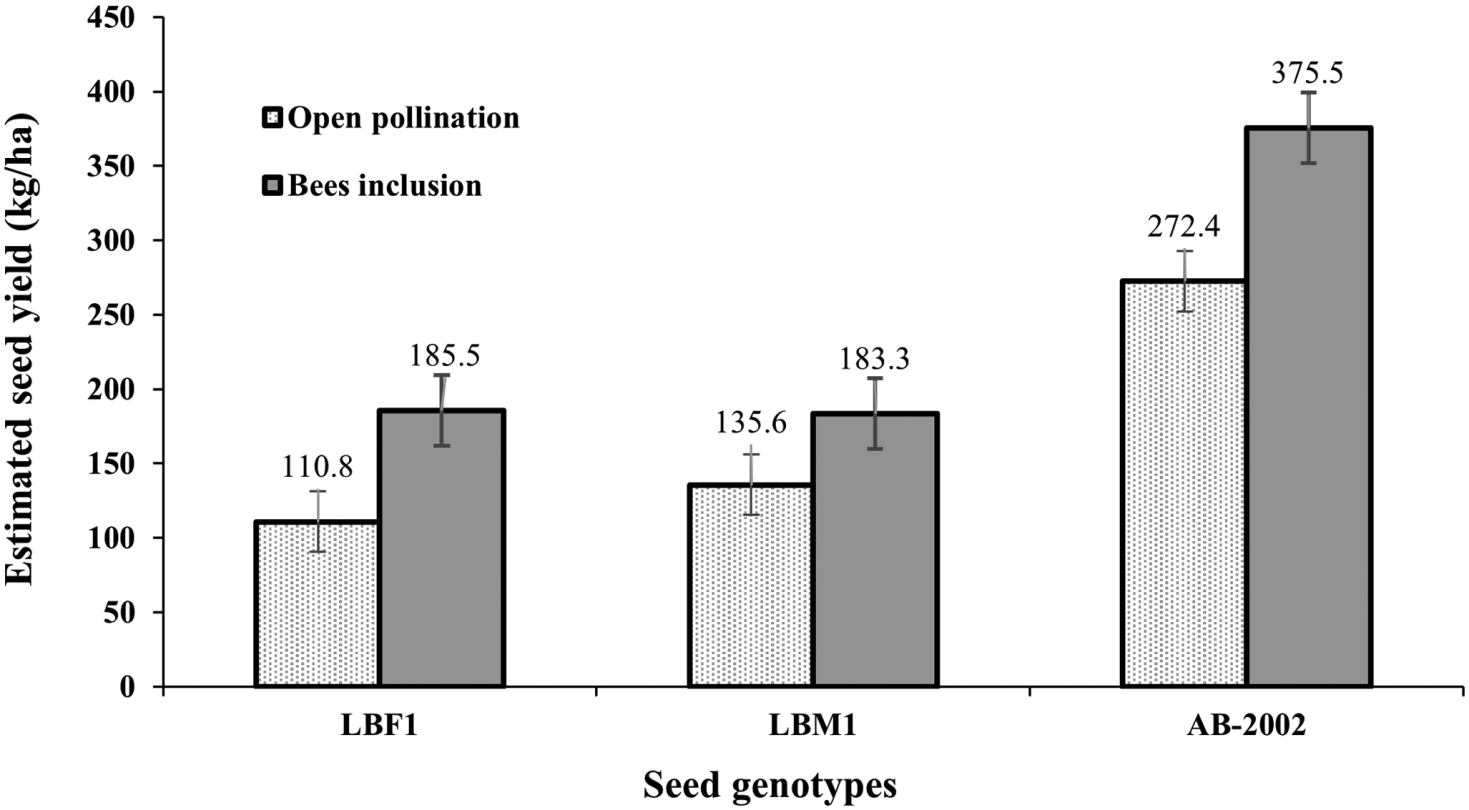
Effects of bees inclusion (honeybee pollination) by *Apis mellifera* L. (Hymenoptera: Apidae) and open pollination on estimated seed yields of 3 berseem clover (*Trifolium alexandrinum* L.) genotypes, i.e., farmer own-saved (LBF1), market (LBM1), and research-station (cv. Agaitti Berseem-2002; AB-2002) seeds.

The impact of the inclusion of honeybees for pollination on 1,000-seed weight varied with genotypes, with an increase for farmer seed (from 2.1 to 2.4 g) and the improved variety (from 3.4 to 3.7 g) (*F* = 25.63; df = 3, 44; *P* < 0.001), but had no effect (*P* > 0.05) with market seed. Honeybee inclusion also resulted in increases in estimated seed yields, regardless of genotype, with an overall increase of 43.5% (ranging between 35% and 67%) over open pollination (*F* = 24.42; df = 3, 44; *P* < 0.001). The maximum (*P* < 0.001) seed yield of 375.5 kg/ha was produced using the improved variety seed and honeybee inclusion (AB-2002 × BI), as shown in Fig. 3. The average seed yields did not vary (*P* > 0.05), between the 2 growing seasons (193.7 vs 227.3 kg/ha).

As shown in Table 3, honeybee pollination treatment increased the estimated seed yield and 1,000-seed weight (*P* < 0.001), as well as number of seeds per head (*P* < 0.05). However, the interaction between pollination treatment × year had no effect (*P* > 0.05) on berseem clover seed production.

**Table 3.** Linear mixed model analysis of pollination treatment, year, and the interaction treatment × year for berseem clover seed yield (kg/ha), seeds per head, and seed weight (g) during 2 growing seasons (2012–2013 and 2013–2014) in central Punjab region of Pakistan

Source of variation	*P* values		
	Estimated seed yield (kg/ha)	Seeds per head	1,000-seed weight (g)
Model	<0.001	<0.05	<0.001
Pollination treatment	<0.001	<0.01	<0.001
Year	0.096	0.506	<0.001
Pollination treatment × Year	0.142	0.025	<0.001

In comparing seed sources/genotypes, honeybee inclusion had the greatest impact (*P* < 0.05) with farmer seed, increasing estimated seed yield by 67.4%, compared to 35.2% and 37.8% for the market and improved variety seeds, respectively (Table 4). The maximum net income of seed was generated from research-station seed in combination with the addition of honeybees [AB-2002 × BI; 168,975 Rs/ha (US$1,728/ha)], while the minimum net income was generated from farmer seed with open pollination [LBF1 × OP; 49,860 Rs/ha (US$510/ha)].

**Table 4.** Impact of insect pollination with the inclusion of honeybees (BI) on increase in seed yields of berseem clover genotypes and net incomes (from seed sale) compared to open pollination (OP) of farmer own-saved (LBF1), market (LBM1), and research-station (cv. Agaitti Berseem-2002; AB-2002) seeds

Treatments	Net seed income (Rs/ha)	Increase in seed yield (%)
LBF1 × OP	49,860 ($510)	–
LBF1 × BI	83,475 ($854)	67.42
LBM1 × OP	61,020 ($624)	–
LBM1 × BI	82,485 ($844)	35.18
AB-2002 × OP	122,580 ($1,254)	–
AB-2002 × BI	168,975 ($1,728)	37.85

## Discussion

The most significant outcome of this study is the 67% increase in seed yield for farmer-saved seed with honeybee inclusion for pollination compared to other seed genotypes, including the research-station cultivar. This substantial improvement highlights the critical role of honeybees as pollinators in enhancing berseem clover seed production at the farm level. Integrating honeybee management into smallholder farming systems can boost forage and seed yields, thereby improving livestock nutrition and on-farm productivity. By adopting these practices, farmers can achieve higher yields and increased incomes, contributing to the sustainability and resilience of smallholder mixed farming systems. Specifically, this integration can bridge the gap between the demand and supply of quality forage seed, directly impacting livestock productivity and farmers’ livelihoods.

Researchers (Roy et al. 2005, Rao and Stephen 2009, Jat et al. 2014a) have reported that higher seed yields in berseem clover are primarily attributed to an increase in seeds per head. These researchers explained this as an indicator of the interaction between genotypes, environment (temperature, humidity, and wind), and pollinators. The present study further supports this, demonstrating that the increase in seeds per head, and consequently higher seed yields across all genotypes, is predominantly due to honeybee pollination.

Being major pollinators, honeybees have an impact on seed setting and ultimately seed yield in berseem clover crop (Jat et al. 2014a). The inclusion of honeybees resulted in increases in all of the other seed yield parameters measured in the present study except the number of heads per m^2^, which were equal to or even greater than other studies involving berseem clover. For example, Dixit et al. (1989) found pollination increased the number of seeds per head by 52%, while Jat et al. (2014a) reported a more moderate response of 6.5% in comparing open pollination (51 seeds/head) to honeybee pollination (55 seeds/head) in their study in Haryana, India. Abdalla et al. (2012) found that excluding pollinators during flowering may result in either none or very poor seed set. The positive effects of honeybees on pollination have also been reported for other legume forage species. For example, Rao and Stephen (2009) found that the inclusion of honeybees for pollination significantly increased the number of seeds per head in red clover (*Trifolium pratense* L.) in Western Oregon, USA. In a recent review of red clover production factors, Shuxuan Jing et al. (2021) emphasized the critical role of insect pollen transfer in seed production, highlighting the often overlooked influence of pollination conditions, including insect populations and climate.

While potential seed size and weight are genetically determined, actual seed size and weight will be influenced by the environment. Heavier and larger seeds are most likely to be influenced by effective pollination and fertilization processes during seed setting (Jat et al. 2014a). The inclusion of honeybees resulted in an 8–11% increase in 1,000-seed weight in the present study. The magnitude of the impact of the inclusion of honeybees on seed weight in the present study was inferior to the 37.1% increase reported by Jat et al. (2014a). However, the severity of prevailing conditions (high temperature) during pollination and seed setting stages were likely to have differed between these studies and this would have a confounding impact on seed development and therefore the seed weight. This increased weight also associated with the genetic potential of the improved variety seed, combined with the potential impact of more effective pollination from honeybee inclusion, would have had a key influence on the seed yields recorded in the present study.

Across all 3 of the genotypes used to grow berseem clover, the inclusion of honeybees resulted in an average 43.5% increase in seed yields. Bakheit (1989) reported a maximum 51% increase in seed yields in response to honeybee pollination. This is lower than the maximum 67.4% increase achieved with farmer seed in the present study, which is significant considering farmer seed is the predominant seed source at the farm level. Moreover, climate change has dramatically altered conditions in the Punjab province of Pakistan in recent years, resulting in a reduction in nesting sites for honeybees and habitat fragmentation (Latif et al. 2014). The altered pollinator assemblage resulting from habitat fragmentation due to land use changes along rural–urban gradients is likely affecting the availability of both nesting places and food resources for honeybees (Hepburn and Radloff 2011, Latif et al. 2014).

### Challenges and implications of honeybee pollination in berseem clover seed production within mixed farming systems

Although natural honeybee populations were not measured in the present study, it can be assumed that it was suboptimal for berseem clover pollination and seed setting as Ahmed et al. (2014) had reported lower (than previous) honeybee populations in the study area. The main factor contributing to these low honeybee populations is the widespread use of neonicotinoid pesticides (Murray et al. 2009). In Pakistan, imidacloprid (a neonicotinoid pesticide) is used extensively, both in the form of spray and as a seed coating, over a range of crops including wheat (*Triticum aestivum* L.), rice (*Oryza sativa* L.), cotton (*Gossypuim hirsutum* L.), maize (*Zea mays* L.), potato (*Solanum tuberosum* L.), and different forages like berseem clover, sorghum (*Sorghum bicolor* L.), and orchards like guava (*Psidium guajava* L.), citrus (*Citrus* spp.), and mango (*Mangifera indica* L.), to protect against sap-feeding insects such as aphids and white fly (Ahmed et al. 2014). Neonicotinoids are toxic and lethal insecticides with LD_50_ (lethal dose kills 50% of the test population) values of 40 and 180 parts per billion (ppb) for oral intake and body contact for honeybees, respectively (Yang et al. 2008). This results in a rapid decimation of populations to the point where effective cross-pollination of crops is not possible (Murray et al. 2009).

A number of factors affect honeybee activity and survival. Both the distance from their nest and time of day influence the frequency of visits (to plants) by honeybees. Jat et al. (2014b) found that the frequency of visits by *A. mellifera* and *A. dorsata* within a 2.5 km flight distance from the hive was positively correlated with the time of day. The maximum number of visits occurred in the morning and the minimum occurred in the afternoon. Furthermore, they have found that the number of visits was influenced by relative humidity and atmospheric temperature. El-Naby et al. (2012) reported the environmental conditions that favored and regulated the movement of honeybees were temperatures of 28–32°C, relative humidity of 45–55%, and a wind speed of 1.2–2 m/s. These conditions lead to an increase in pollination efficiency, seed setting and thus seed yield of berseem clover. In contrast, Ismail et al. (2013) reported no relevance of the relative humidity on the activity of honeybees.

As shown in Fig. 3, the response to honeybee pollinators can vary, depending on the particular variety/cultivar of berseem clover grown. Abdalla et al. (2012) and Meena et al. (2015) found that in multi-cut berseem clover varieties (as used in the present study), the varieties with a later flowering time (coinciding with warmer temperatures and higher pollinator activity), produced the highest seed yields with greater seed setting percentages compared to those varieties with early flowering times (blooming dates) due to cold winter weather conditions. However, differences in flowering time do not account for the differences in seed yields in response to honeybee inclusion in the present study. There was no difference in the number of heads per m^2^ of the 3 genotypes used, indicating that they flowered at the same time. Seed yield differences in the present study were therefore not a consequence of stem production within the genotypes but were associated with the development of viable seeds (Abdalla et al. 2012).

**Fig. 4.**
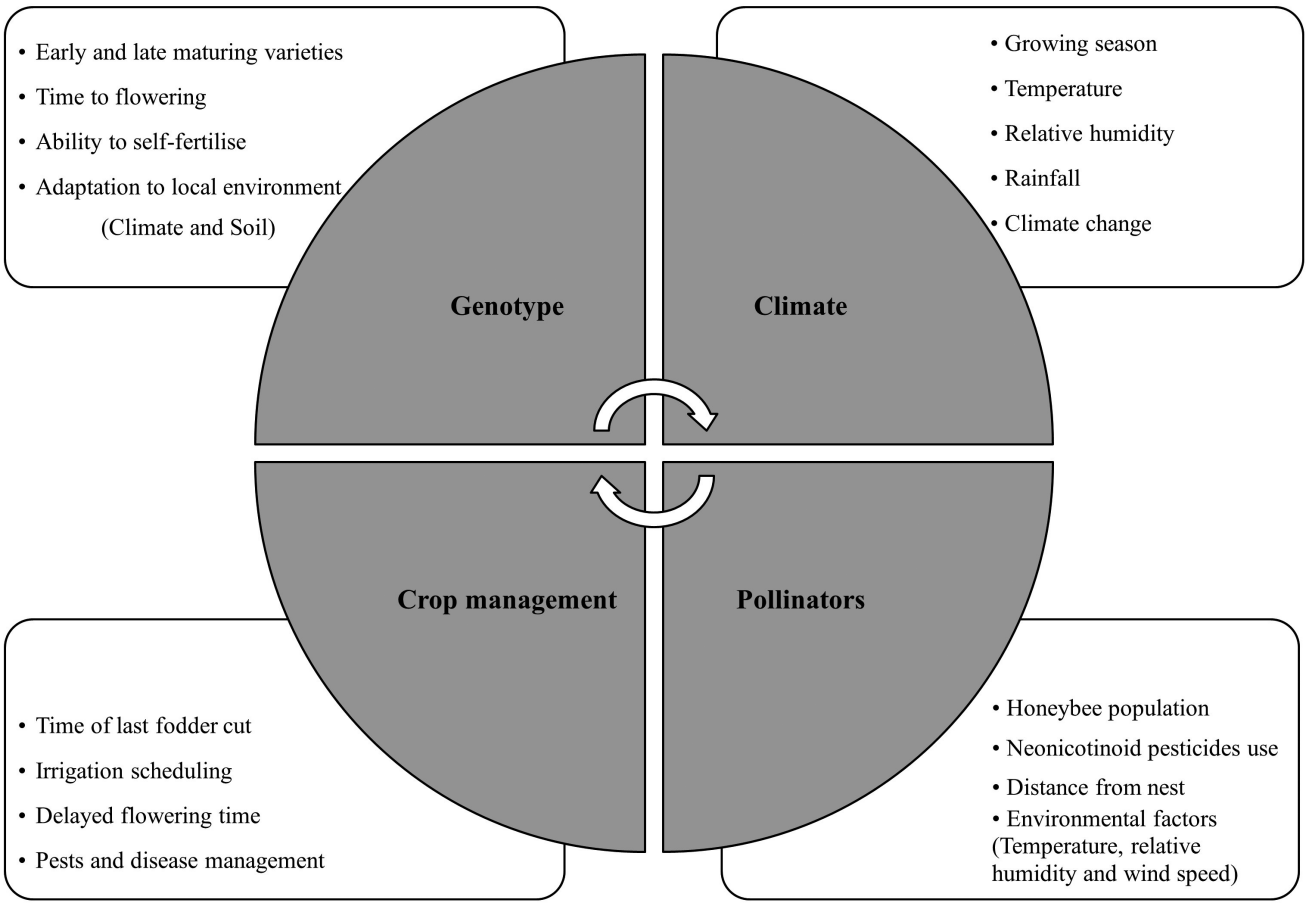
Genetic, environmental, pollinator, and crop management factors affecting the successful pollination process and seed setting in berseem clover.

The intricate interplay between pollination mechanisms and various abiotic (temperature, humidity, and wind) and biotic (genotypes, floral resources, and insects/pollinators) factors, as reported by several researchers (Thapa 2006, El-Naby et al. 2012, Ismail et al. 2013, Latif et al. 2014, Jat et al. 2014b, Meena et al. 2015, Harris and Ratnieks 2024), has limited our comprehension of pollination processes in seed-producing crops. Figure 4 provides a visual representation that aids in understanding this complex intricate relationship. Under the agro-climatic conditions of the central Punjab region of Pakistan, our study demonstrates the positive impacts of increasing honeybee populations in proximity to the berseem clover crop. However, managing these insect vectors remains a significant challenge in the mixed farming systems operating in the study area. Furthermore, climate change may also be affecting the number of honeybees and their pollination efficiency by migration of honeybee colonies between Punjab and Khyber Pakhton Khawa provinces of Pakistan (Hepburn and Radloff 2011). Harris and Ratnieks (2024) suggest that increased use of improved cultivars of red clover in agricultural systems can provide more floral resources for pollinators during its bloom, benefiting both farmers and pollinators.

### The impact of optimizing genotypes and honeybee pollination for economic sustainability in smallholder mixed farming systems

The quantitative yield produced by berseem clover genotypes is the ultimate reflection of the positive relationship with pollinators, which is crucial for effective pollination, fertilization, and seed setting. Higher seed yields result in increased income for seed growers (farmers). Compared to open pollination, the inclusion of honeybees resulted in increased seed yields of 35%, 67%, and 38% for market seed, farmer seed, and improved variety seed, respectively. As a consequence of honeybees’ inclusion, estimated net seed incomes for market, farmers, and research-station cultivars would increase by 21,465 Rs/ha (US$220/ha), 33,615 Rs/ha (US$344/ha), and 46,395 Rs/ha (US$475/ha), respectively (Table 4). These economic incentives are crucial for encouraging the adoption of improved pollination practices and the use of high-yielding research station seed varieties.

The use of improved variety seed with the inclusion of honeybees produced the highest berseem clover seed yields (375.5 kg/ha). The estimated maximum net income of seed (168,975 Rs/ha; US$1,728/ha) was from the improved variety seed with (supplemented) honeybee pollination, while the minimum net income (49,860 Rs/ha; US$510/ha) was achieved from farmer seed with open pollination, the current practice of smallholder farmers. These changes in net incomes are in agreement with those of Goodman and Williams (1994), who reported an increase in net income of US$654/ha with the inclusion of additional honeybees in white clover in Melbourne, Australia as a result of increased seed yield over an operating loss of US$ –109/ha with no pollination (no access by honeybees all the time). The inclusion of (additional) honeybees as pollinators would increase income to the farmers, which is of particular significance in the context of smallholder farmers. In addition, the potential still exists for further increase in net income through the sale of honey both in the domestic and export markets, and thus further potential financial benefits to the farms can be established. The increase in net income would come not only through increased seed yields but also (potential) sale of honey. Production and sale of honey would be considered as potential opportunity in the study area and can further increase profit margins of smallholder farmers. However, honey production and sale were not measured in the present study.

Utilizing improved cultivars that produce more inflorescences, coupled with better harvesting management in terms of timing and frequency of cutting forage crops, could significantly increase floral density in various cultivars, including local landraces (seed produced and exchanged between farmers), which would be beneficial in creating a potential win-win situation for both farmers and pollinators (Harris and Ratnieks 2024). Assessing these traits across cultivars is straightforward and can be achieved through visual assessments, surveys, or joint experimentation with farmers (participatory research) to quickly quantify the number of florescence and seed heads per unit area (Tufail et al. 2020). Providing this information to farmers can be relatively simple and cost-effective for research institutes and seed companies. Furthermore, farmers can witness firsthand, in a test planting similar to the one in this study, the varying number of seed heads produced by different cultivars, such as farmer own-saved, market, and the improved variety cultivars.

The present study demonstrates that there is immense scope for increasing seed yields, not only by using improved varieties but also by increasing the honeybee population in the vicinity of berseem clover fields. Further improvements to income may be derived from higher quality seed which may occur with the larger seeded research-station improved variety seed. Differences in net incomes presented here are likely to be a maximum given honeybee populations in this study were considerably greater than what could be achieved in the field, however, when coupled with other gains likely from such agronomic and genetic modifications, greater farm incomes may be achievable.

The findings of this study have significant implications for informal farmer-collected seed production systems. By demonstrating the substantial yield benefits of honeybee pollination, farmers are encouraged to manage and include honeybee populations in their forage production systems. This practice not only boosts seed yields but also improves forage quality, enhancing livestock nutrition and on-farm productivity. Integrating honeybees can enable research stations to achieve higher seed yields and better-quality seeds, which can be distributed to farmers, thereby strengthening the current formal seed distribution system. This integration fosters the adoption of honeybee management practices and improved seed cultivars, providing significant benefits for smallholder farmers, research stations, and the agriculture sector as a whole. Consequently, these sustainable practices significantly enhance overall farm productivity and resilience.

While this study provides valuable insights into the benefits of honeybee pollination for berseem clover seed production, it has a few limitations. The experimental design allowed honeybees to choose which cultivar to visit, introducing a potential bias. Furthermore, experimental conditions may not reflect real-world farming scenarios, and associated factors (social or economic) influencing the adoption of these practices were not considered. In addition, the direct effects of meteorological factors such as rainfall, temperature, and relative humidity on pollination efficiency were not measured. This study focused exclusively on honeybees, excluding other potential pollinators, and did not account for long-term climatic variation. Although the economic benefits were highlighted, a detailed cost-benefit analysis was lacking. Future research should focus on optimizing pollination management strategies to address these existing gaps, and exploring additional benefits including honey production and honeybee impacts on crop quality, to further enhance the economic viability of smallholder mixed farming systems.

Collectively, the results of the present study suggested that honeybees play an important role in increasing seed yields of berseem clover. For all the genotypes of berseem clover used, the estimated seed yield and its associated yield parameters were improved by introducing a supplemental (netted) population of honeybees. Consequently, a doubling (119% increase) in the seed yields by using improved variety seed in combination with honeybee inclusion compared to farmer seed in open pollination was achieved. Furthermore, the seed income would also be increased with an additional net income of 46,395 Rs/ha (US$475/ha). Taken together, our results suggest that the use of improved variety seeds along with the integration of honeybee pollinators can substantially boost berseem clover seed production and enhance economic returns for smallholder farmers.
